# Thyroid dose assessments due to inhalation of ^131^I for nuclear medicine workers

**DOI:** 10.3389/fpubh.2022.1027782

**Published:** 2022-12-05

**Authors:** Gang Liu, Ye Li, HanYu Zhang, Xue Zhang, Yin Yin Liu, Xiao Qin Wu, Li Mei Niu, Rong Zhang

**Affiliations:** Gansu Province Center for Disease Control and Prevention, Joint Laboratory of Institute of Radiology, Chinese Academy of Medical Sciences, Lanzhou, China

**Keywords:** ^131^I, thyroid, radiation protection, internal doses, nuclear medicine

## Abstract

**Background:**

In general, medical staff who work in nuclear medicine should be entirely safe in their professional environment. Nevertheless, we already know that the working environment of the nuclear medicine staff is not completely safe due to the handling of high amounts of radionuclides for diagnostic and therapy applications, which is especially relevant for ^131^I (as a non-sealed source).

**Purpose:**

The goal of this study was to assess the inhaled ^131^I thyroid dose in nuclear medicine workers and to introduce a simple method for internal exposure monitoring.

**Methods:**

Using 2-IN^*^2-in NaI (Tl) scintillation spectrometer and its supporting software (InSpector Maintenance Utility and Genie 2000), from 2019 to 2021, internal thyroid irradiation monitoring, an internal thyroid irradiation monitoring investi A NaI (Tl) scintillation spectrometer and its sugation was carried out for 3 consecutive years, between 2019 and 2021, in staff members of nuclear medicine departments engaged with iodine therapy.

**Results:**

^131^I activity was found highest in the thyroid of nuclear medicine workers involved with the manual packaging and delivery of the radioisotope, while it was not detected in staff members involved with the automatic packaging and drug delivery. The activity range was found to be 30.00 ± 6.60–6070.00 ± 1335.40 Bq for the exposed personnel, and the estimated dose was 0.05–6.77 mSv. In 2021, three workers had an annual equivalent dose above 5 mSv.

**Conclusion:**

By monitoring the thyroid ^131^I in staff members of the nuclear medicine department, it was found that there are ^131^I internal occupational exposure risks. The best solution is automatic packaging and drug delivery.

## Highlights

1. In this study, a simple monitoring method was established to measure the dose derived from ^131^I in the thyroid glands of nuclear medicine staff in normal working conditions.
2. This study provides theoretical and data support for formulating radiation protection policies and standards in China.

## Introduction

Iodine is one of the essential elements for the normal functioning of the human body. It is also the most widely used radioactive isotope in nuclear medicine and is widely used in the diagnosis (^123^I, ^125^I, and ^131^I) and treatment (^125^I and ^131^I) of various diseases. ^131^I is an important radionuclide in nuclear accidents. For example, after the Chernobyl accident in 1986, the contamination of ^131^I (and other short-lived isotopes such as ^132^I and ^133^I) caused an increase in the incidence of differentiated thyroid cancers in children but not in adults. The younger the age, the higher the incidence (1–5).

Sources of radiation in nuclear medicine are primarily radiopharmaceuticals, which are invisible and ubiquitous; it is common in patients, excrement, and discarded medical equipment. These factors bring certain difficulties to radiological protection in nuclear medicine processes and procedures of nuclear medicine. In recent years, medical cyclotron and PET equipment used for the production of positron-emitting drugs have been gradually introduced. This has brought new problems to the radiation protection of the nuclear medicine staff.

The issue of internal radiation exposure in nuclear medical personnel is of great concern internationally, and its research mainly includes the determination of internal radiation monitoring in nuclear medical personnel.

In recent years, the development of clinical nuclear medicine has increased the risk of potential radiation exposure and environmental pollution for the professional population and the public. With in-depth research on the effects of radiation on cells, more attention has been paid to radiation protection.

In occupational exposure protection, one of the current focuses is the occupational exposure monitoring of nuclear medicine workers and the prevention and treatment of occupational radioactive diseases (6).

In recent years, nuclear medicine has developed rapidly, and the diagnosis and treatment with nuclear medicine have shown a trend of rapid growth. As a result, the level of exposure of medical personnel has increased significantly in recent years. At the end of 2009, China established a national central database for individual monitoring of radiation workers, which has steadily increased the coverage of individual monitoring of external exposure. By the end of 2019, the database had covered 43,413 employers in radiation work, with 395,040 people monitored.

According to a national survey on the status of nuclear medicine conducted every 2 years by the Chinese Society of Nuclear Medicine, the number of specialized units engaged in nuclear medicine in 2020 was 1,148, an increase of 33% over 2002, and the number of personnel engaged in nuclear medicine-related work increased from 5,600 in 2002 to 12,578 in 2020 (7–11). Therefore, there is an urgent need to improve the monitoring of individual doses by nuclear medicine practitioners, as radiation prevention and control measures and occupational health monitoring based solely on external exposure measurements are not sufficient. In contrast, internal exposure monitoring in most provinces and cities in China has not been carried out effectively. In consideration of practical and economic factors, China should carry out comprehensive research on internal radiation monitoring and dose estimation in nuclear medicine, appropriately develop and verify internal radiation dose calculation and evaluation software, and establish an individual dose monitoring system for internal radiation. In addition, to expand and update limited information on trends in the average annual dose and time of exposure within nuclear medicine, workers should propose targeted and operational prevention and control measures.

Radiation protection in clinical nuclear medicine diagnosis and treatment has aroused widespread concern in society with the increase in diagnosis and treatment equipment and patients. It is an urgent problem to improve the safety awareness and protection level of radiation workers in the clinical application of nuclear medicine.

In this article, a simple monitoring and evaluation method for ^131^I in the thyroid glands of nuclear medicine staff was introduced in detail. In 3 consecutive years from 2019 to 2021, the thyroid activity of ^131^I was measured in members of nuclear medicine departments.

## Materials and methods

### Object of study

In 3 consecutive years, from 2019 to 2021, employees from 4 nuclear medicine hospitals among the highest-rated general hospitals in Gansu Province were monitored. Table 1 reports the data on the staff members subject to the study.

**Table 1:** Information on radiological staff working on iodine therapy in the nuclear medicine branch.

**Subject**	**Sex**		**Profession**		
	**Male**	**Female**	**Nurse**	**Technician**	**Physician**
2019	9	11	3	5	12
2020	18	20	8	12	18
2021	15	17	7	11	14

### Methods of measurement for ^131^I

A method of prompt and rapid estimation of thyroidal ^131^I exposure dose of the nuclear workers was performed with a 2-in^*^2-in NaI (Tl) scintillation spectrometer and its accompanying software for energy spectrum analysis (InSpector Maintenance Utility and Genie 2000).

### Survey research

Each participant was asked to complete a questionnaire and fill out an informed consent form. The first questionnaire is in the category of “demographic”, which contains general questions on gender, age, affiliation, workplace, etc. The second questionnaire mainly focuses on the nature of the work and ^131^I treatment, the duration of exposure to ^131^I, and details of the contact with patients after treatment or diagnosis. The third questionnaire addresses the health status of thyroid disease.

### Measurement requirements

Meeting rooms and other areas with low background radiation (<200 nGy/h) were used to conduct the measurement and avoid radioactive contamination. We record the basic personal information and operation nuclide information of medical institutions and monitored personnel. The instrument probe was wrapped with clean plastic tape (or plastic film). In order to prevent contamination of the instrument probe, each measurement is replaced in time (including the measurements of the thigh and thyroid of the same person, which also need to be replaced) to prevent contamination of the instrument probe. If the subject was holding the probe, disposable gloves had to be worn. The subject sits on an adjustable height chair, the portable digital spectrometer is glued to the thyroid and the thigh for measurement, and the measurement time is 120 s. For the same subject, the thigh and thyroid were measured once, the spectral file name of each thigh and thyroid test was recorded, and detailed records were made on the information sheet. The measured values on the thigh were used as a background evaluation.

### Calibration of the monitoring instrument

The monitoring instrument is calibrated by the National Institute of Metrology, China, and the National Institute for Radiological Protection, Chinese Center for Disease Control and Prevention (NIRP, China CDC). The ^131^I serves as the calibration source for the 2-in^*^2-in NaI (Tl) scintillation spectrometer energy calibration. The measurement time was 120 s, the calibration coefficient was 9.4 × 10^−3^ cps/Bq (cps is count per second), and the MDA (minimum detectable activity) was 19.0 Bq. NaI measurement ranges are 50 keV~3 MeV, 10.0 nSv/h~100 mSv/h, and 10.0 nSv−10.0 Sv probe. The ^131^I is a standard source with an emission energy of 364.5 keV and a half-life of 8.04 days, as shown in Table 2.

**Table 2:** ^131^I standard source and percentages of uncertainty.

**Nuclide**	**Activity (Bq)**	**Energy (keV)**	**Branching ratio**	***T*****_1/2_** **(d)**	**Original activity date - date of measurement (d)**	**Net**	**Uncertainty**	***T*** **(s)**	**Efficiency value**	**cps**
I-^131^	3.311 × 10^4^	364.5	0.817	8.04	55.4	2,263	8.9%	120	8.3 × 10^−2^	18.6
I-^131^	3.311 × 10^4^	364.5	0.817	8.04	55.4	1,250	16.9%	120	4.6 × 10^−2^	10.4
I-^131^	3.311 × 10^4^	364.5	0.817	8.04	55.4	1,513	14.0%	120	5.6 × 10^−2^	12.6
I-^131^	3.311 × 10^4^	364.5	0.817	8.04	55.4	1,354	15.7%	120	5.0 × 10^−2^	11.3
I-^131^	3.311 × 10^4^	364.5	0.817	8.04	55.4	1,693	12.6%	120	6.23 × 10^−2^	14.1
I-^131^	3.311 × 10^4^	364.5	0.817	8.04	55.4	1,940	10.6%	120	7.14 × 10^−2^	176.8
I-^131^	3.311 × 10^4^	364.5	0.817	8.04	55.4	1,147	18.3%	120	4.2 × 10^−2^	9.6
							13.8%		5.9 × 10^−2^	

### Efficiency calibration

The portable spectrometer is connected to the computer, and the efficiency scale is completed by Genie 2000 spectral analysis software. Accurate efficiency calibration is the basis of quantitative measurement. In this monitoring, the calibration experiment was carried out using the IAEA neck module (the International Atomic Energy Agency (IAEA)/American National Standards Institute (ANSI) thyroid calibration neck phantom), thus, the efficiency scale factor epsilon is obtained. The calibration source is ^131^I. The general procedure for the efficiency scale is as follows. The system parameters are adjusted to have the spectrometer in a stable and good working state, and the condition should be in conformity with the measurement conditions, such as the measuring interval of timeand the distance between the detector and the measuring target.

It is generally required that the statistical error of the minimum net peak area count be <0.5% (2σ). If necessary, the measurement is repeated 2–3 times. Using the same spectral analysis method in the monitoring measurement, we calculate the net area count of 364.5 keV characteristic peaks in the calibration source spectrum after removing the Compton scattering signal interference.

Then, we calculate the efficiency scale factor of the energy of the gamma ray generated at 364.5 keV CF_s_, as follows:


(1) $$\begin{array}{l} CF_{s}=\frac{n_{s}}{A\cdot e^{-\lambda \Delta t}} \end{array}$$

In the formula:

CF_s_–Instrument efficiency scale factor;
N_s_–IAEA neck module 364.5 keV total peak net area count rate;
A—The activity of the ^131^I calibration source at the time of preparation or the constant value (t_0_);
λ —The decay constant of the nuclides (s^−1^);
Δt —The decay time of the source in the time interval between the source preparation time or the time of fixed value and the start of the measurement (d). *e*^−Δ*t*^ is the decay correction factor for this interval of time.

### Dose estimation

#### Data analysis

Formula (2) is adopted for the calculation of Aj:


(2) $$\begin{array}{l} A_{j}=\frac{\left(n_{j}-n_{b}\right)}{{CF}_{s}\cdot e^{-\lambda \Delta t}} \end{array}$$

In the formula:

*A*_j_–Measurement activity of radionuclide iodine-131, Bq;
*n*_j_–Thyroid 364.5 keV total peak net area count rate was measured, s^−1^;
*n*_b_–Thigh 364.5 keV total peak net area count rate was measured, s^−1^;
  _CF*s*_–Instrument efficiency scale factor from the efficiency scale process;
  λ—The decay constant of the nuclides (s^−1^), according λ = ln2 / *T*_1/2_, *T*_1/2_ is the physical half-life of ^131^I (8.0 days);
Δt—Time interval (s) between the completion of the last operation ^131^I and the start of this thyroid measurement. *e*^−Δ*t*^ is the decay correction factor for this period.

### Data report

#### Expression form of thyroid activity measurement results

If the thyroid activity measurement result is greater than the detection threshold, it is expressed as “measurement activity result ± extended uncertainty (reference time)”, and the uncertainty is expressed with at most 2 significant figures.

If the MDA is lower than the detection limit of the instrument, we use “ <MDA” “instrument detection lower limit”. This means that the 1/2 detection lower limit should be recorded when occupational exposure dose statistics are needed.

At 95% confidence, the 2-in^*^2-in NaI(Tl) scintillation spectrometer uses Formula (3) to calculate the instrumental detection limit MDA for ^131^I at a given measurement time.


(3) $$\begin{array}{l} MDA=\frac{2.706+4.653\sqrt{N_{b}}}{CF_{S}\cdot t_{m}} \end{array}$$

In the formula:

*MDA*—Minimum detectable activity, in the unit of Becqurel (Bq);
*N*_b_–The 364.5 keV background count of the total absorption peak of γ rays, which includes the interference peak count (if any) caused by the nuclides in the detector and its surrounding environment and the contribution of the continuum spectrum of other high-energy γ emitters in the measurement;
*CF*_s_–The efficiency scale factor of the instrument, from the efficiency calibration process;
*t*_m_–Measuring time (s).

### Extended uncertainty of measurement results

#### Combined standard uncertainty

Each component of the measurement result uncertainty includes using a class A or class B evaluation method to get the component; class A method refers to the method calculated by the Bessel formula through multiple measurements; class B methods refer to non-class A evaluation methods, such as the uncertainty of the nuclide activity contained in the calibration source, which is generally directly quoted from the calibration source certificate. Each uncertainty component u_i_ is synthesized by the “square and root” method to obtain the synthetic standard uncertainty u_c_, which is calculated by Formula (4):


(4) $${\text{u}}_{\text{c}}=({\text{u}}_{{\text{1}}^{2}}+{\text{u}}_{{\text{2}}^{2}}+\ldots {\text{u}}_{{\text{n}}^{2}}{)}^{1/2}$$

In the formula:

u_c_–Combined standard uncertainty;
u_n_–Each uncertainty component (u_1_,u_2_,…,u_n_), generally including counting statistical uncertainty, scale source uncertainty, and geometric position uncertainty. In the formula: scale source uncertainty is 5.6%; relative error is <10%; and geometric position uncertainty can be ignored (when specifying a routine monitoring program, it is generally assumed that work processes and workplace conditions do not vary greatly over time).

$$\begin{array}{l} u_{c}=0.11 \end{array}$$

#### Expanded uncertainty

The extended uncertainty U is calculated by Formula (5):


(5) $$\begin{array}{l} U=k\times u_{c} \end{array}$$

In the formula:

U—Expanded uncertainty;
k—Include factor, generally 2, the corresponding confidence is about 95%.
u_c_ —Combined standard uncertainty.

$$\begin{array}{l} U=0.22 \end{array}$$

#### Accumulated dose of thyroid organs

The time of thyroid intake of ^131^I is calculated according to the time of completion of the last operation. The accumulated dose in the thyroid calculated using Formula (6) and (7) is adopted for calculation.

The formula for calculating thyroid intake:


(6) $$\begin{array}{l} I=M/m\left(t\right) \end{array}$$

In the formula:

*I*—The radionuclide intake (Bq);
*M*—The radioactive activity (Bq) measured *in vivo* or in organs at *t* days after ingestion, namely, Aj in Formula (2);
*m*(*t*)—The radioactivity in the body or organ at *t* days after ingestion (Bq/Bq).

The specific days of the last operation of ^131^I by the nuclear medicine staff were determined through the basic investigation, and different *m*(*t*) values were selected according to the relevant monitoring requirements of GBZ129 (12).

The formula of effective dose to be accumulated:


(7) $$\begin{array}{l} E_{\left(\tau \right)}=I_{\text{jp}}e_{\text{jp}}\left(\tau \right) \end{array}$$

*E*(τ)—The unaccumulated effective dose (Sv);
*I*_jp_–The intake of class J nuclides through the Class P pathway (Bq), namely, I in Formula (3);
*e*_jp_ (τ)—The dose coefficient (Sv/Bq) of class J nuclides through the Class P pathway.

The main cause of internal exposure for nuclear medicine workers is the inhalation of radioactive aerosols and gases during the leaching and packaging of ^131^I, whose chemical form is f. The median aerodynamic diameter (AMAD) of ^131^I aerosols is 5 μm, and the dose coefficient is 1.1 × 10^−8^ Sv/Bq. In this investigation, the monitoring period was set at 30 days according to the workload, and the annual effective dose to be accumulated was calculated.

## Results

**Figure 1:**
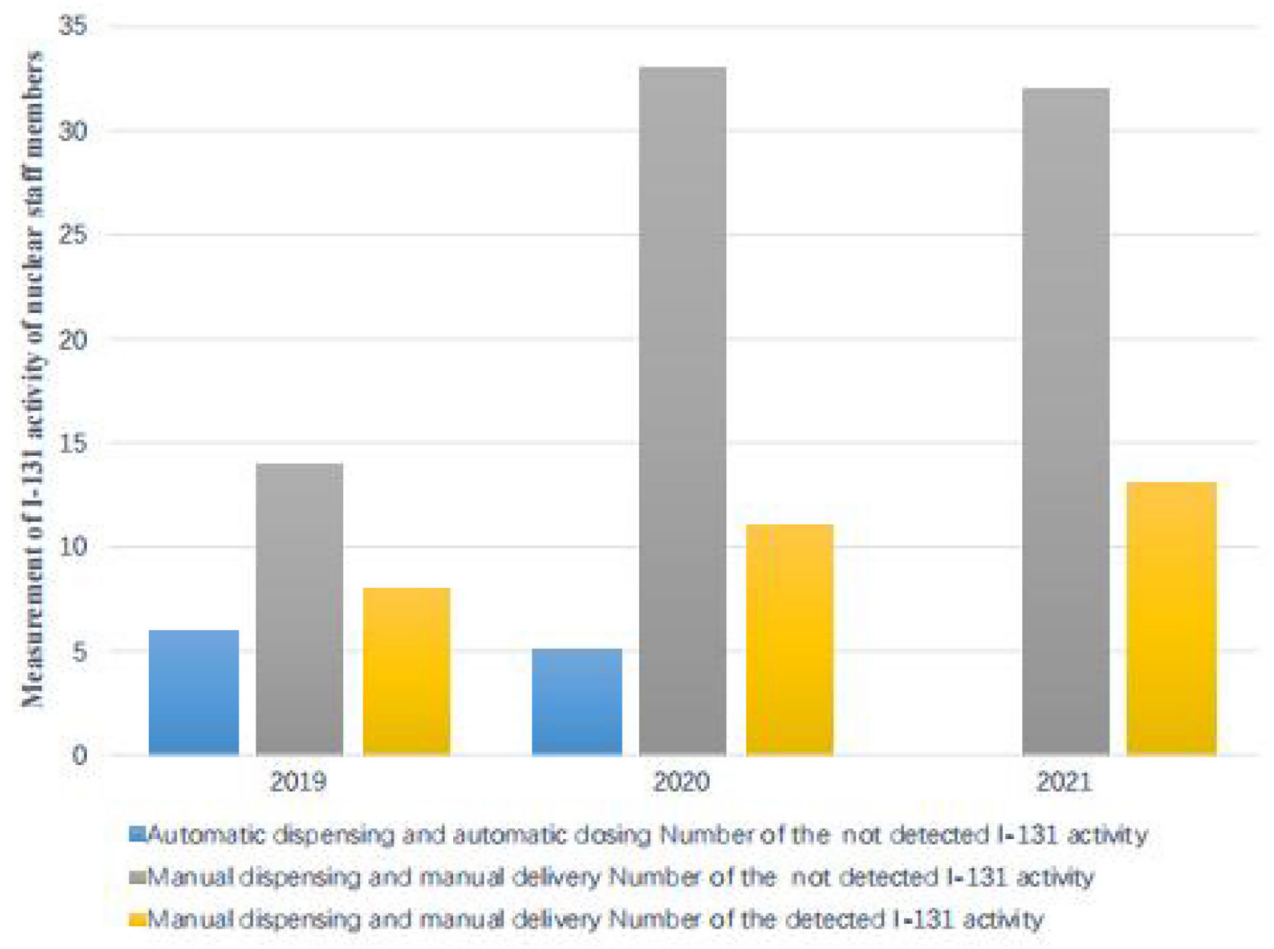
Measurement of ^131^I activity in the thyroid of nuclear medical staff members under investigation.

**Figure 2:**
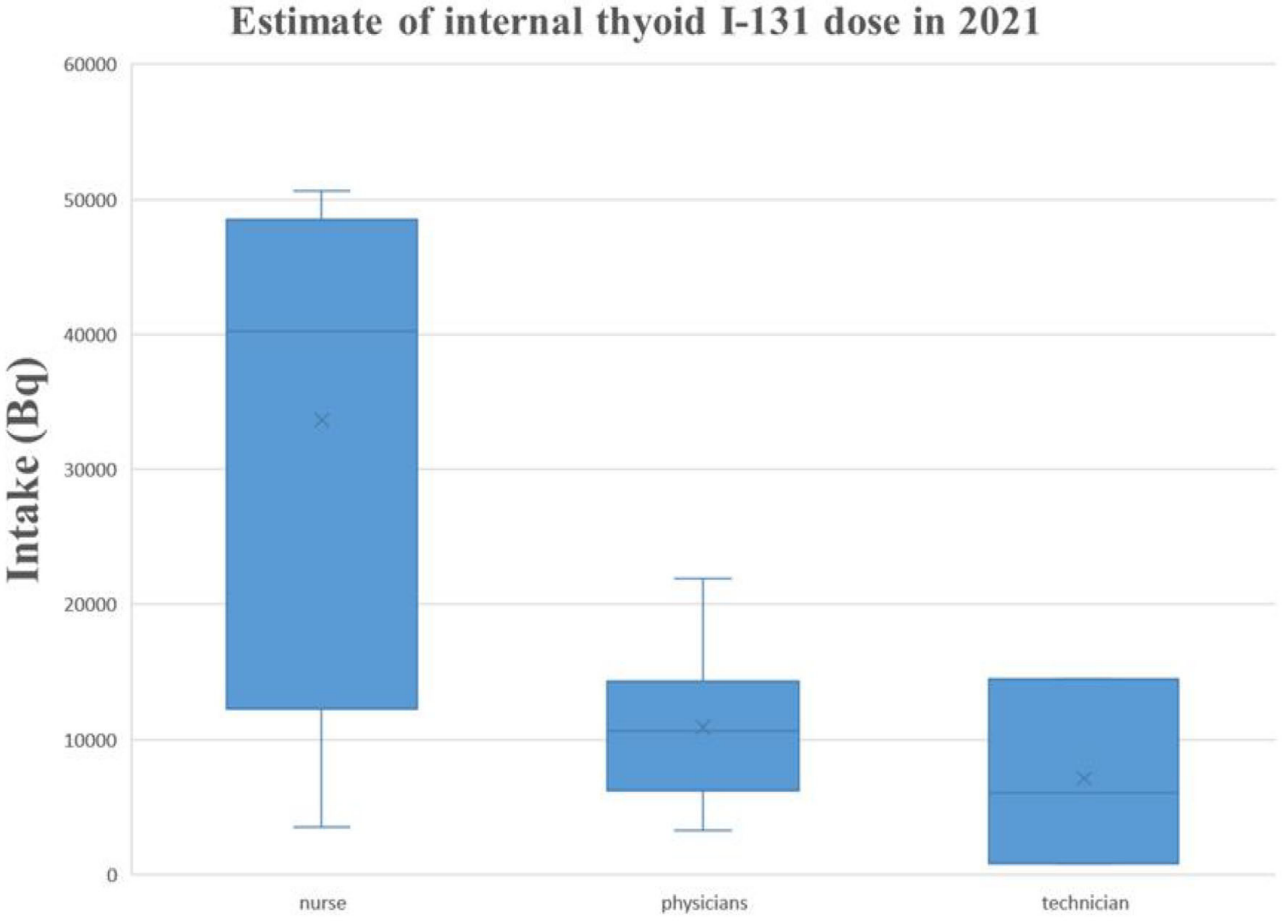
Estimate of internal thyroid ^131^I intake (Bq) in 2021.

**Figure 3:**
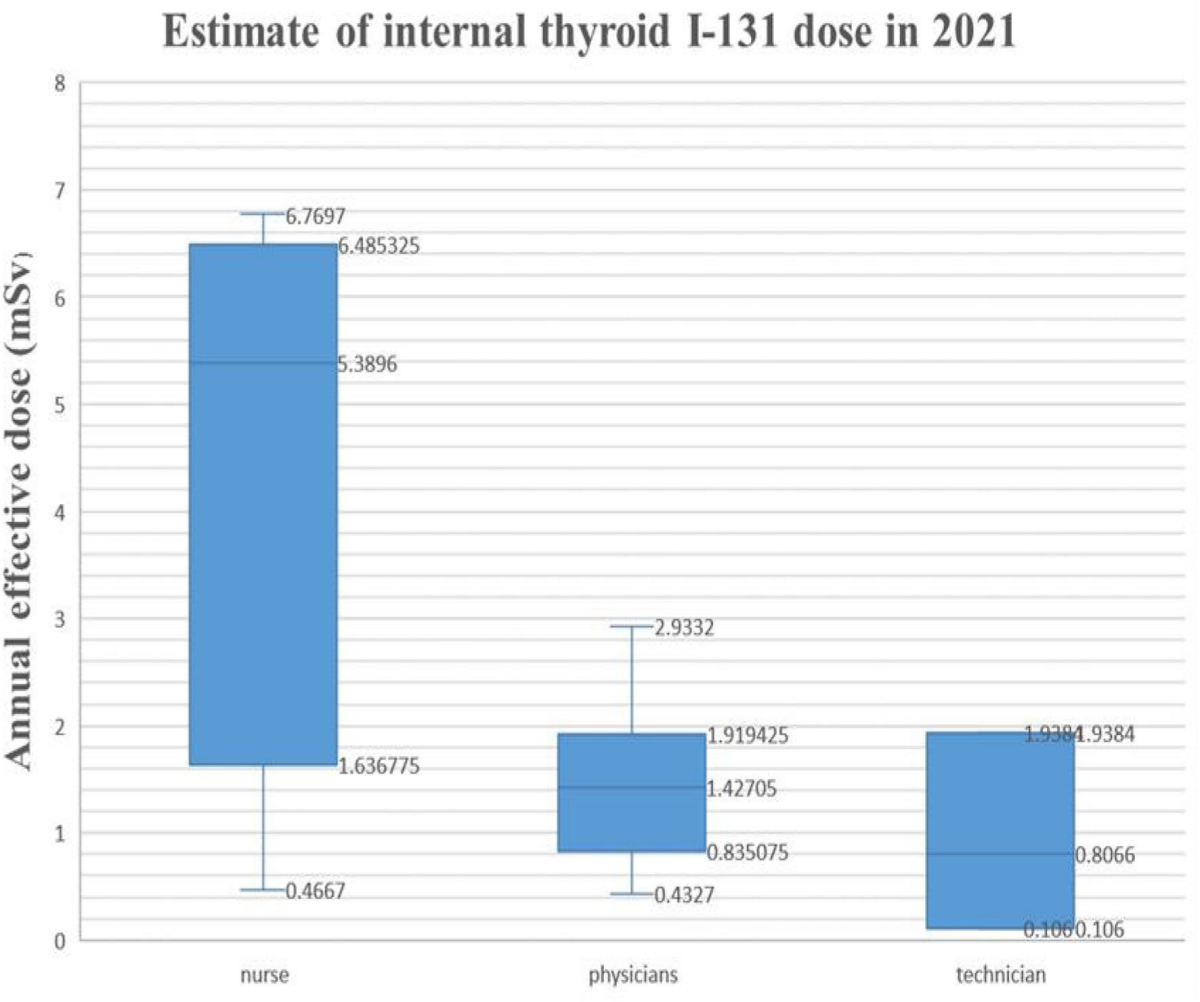
Estimate of internal thyroid ^131^I annual effective dose (mSv) in 2021.

**Figure 4:**
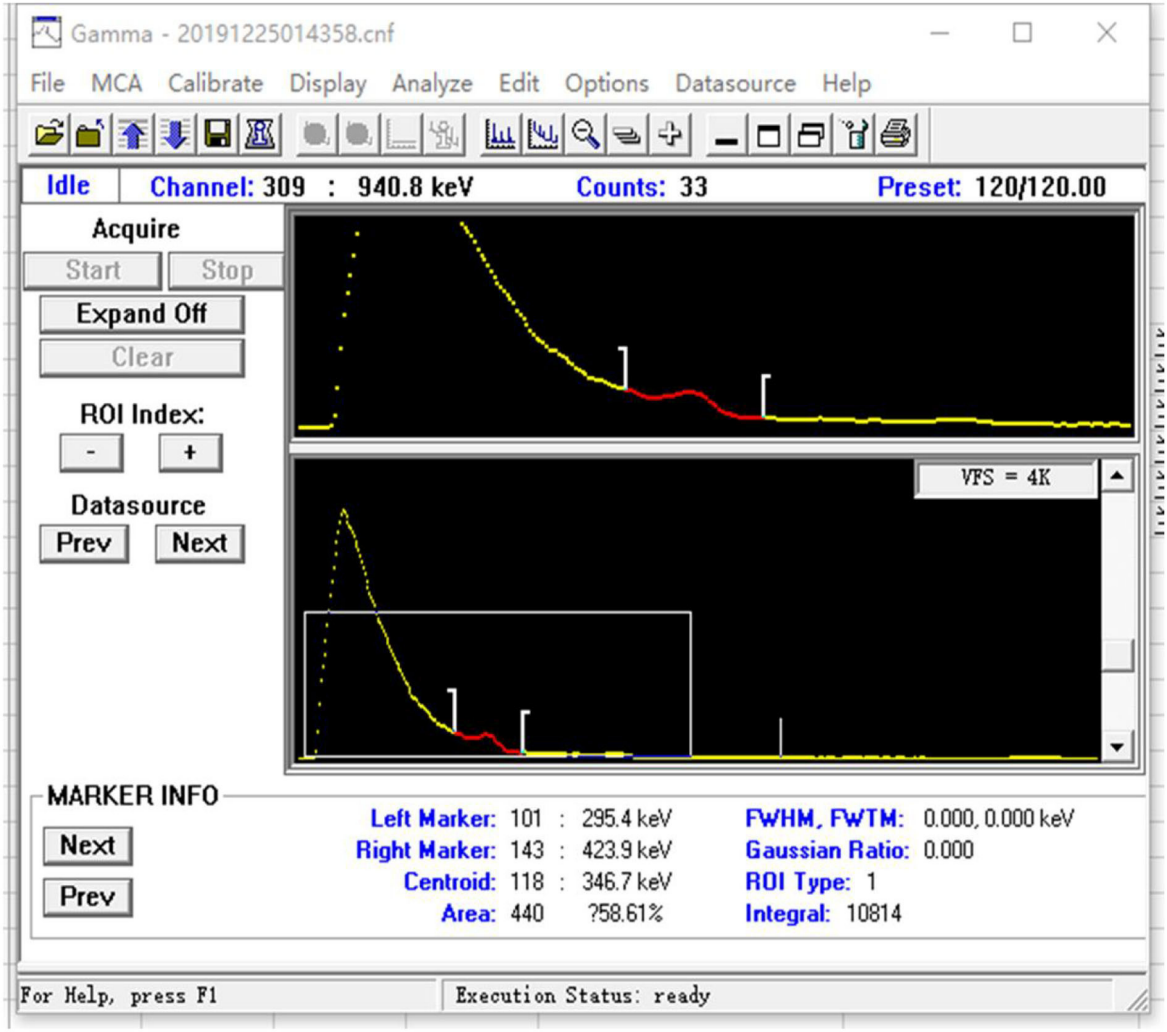
(Background) Energy spectrum diagram.

**Figure 5:**
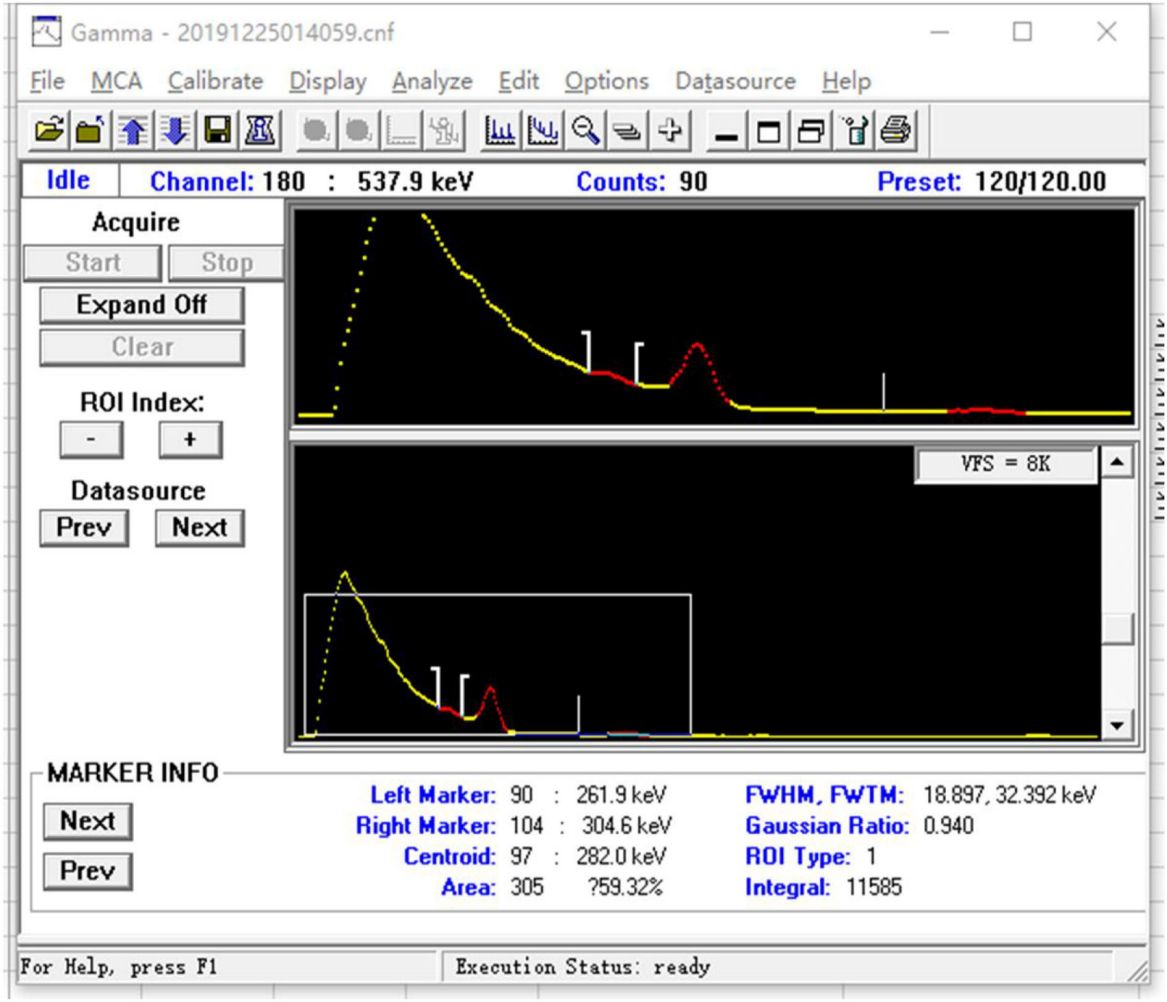
(Thyroidea) Energy spectrum diagram.

In this study, in 3 consecutive years, from 2019 to 2021, the thyroid activity of ^131^I was measured in members of nuclear medicine departments. Those who detected thyroid iodine activity were nuclear medicine workers with manual packaging and manual administration. No thyroid iodine activity was detected by nuclear medicine staff members using automatic packaging and drug delivery. The detection rate of manual packaging and manual administration was compared with the detection rate of thyroid iodine activity for automatic packaging and automatic administration. The difference was statistically significant (*P* < 0.01). In 2019, among the 20 nuclear medicine workers examined, 4 workers were found with a ^131^I activity higher than the detection limit (MDA = 19.0 Bq of ^131^I in the thyroid). The activities that were measured ranged from 32.00 ± 7.04 Bq to 1192.00 ± 262.24 Bq. In 2020, among the 38 nuclear medicine workers examined, 9 workers were found with a ^131^I activity higher than the detection limit (DL = 5.0 Bq of ^131^I in the thyroid). The activities that were measured ranged from 65.00 ± 14.30 Bq to 440.00 ± 96.80 Bq. In 2021, among the 32 nuclear medicine workers examined, 13 workers were found with a ^131^I activity higher than the detection limit (MDA = 19.0 Bq of ^131^I in the thyroid); the estimated dose is 0.05–6.77 mSv. According to the GBZ 128, specifications for individual monitoring of occupational external exposure (7), in 2021, the annual dose of ≥5 mSv was 3 (9.4%). The activities that were measured ranged from 70.90 ± 15.60 Bq to 6070.00 ± 1335.40 Bq. For detailed results, Tables 3–6 report the data, and Figures 1–5 are the monitoring results.

**Table 3:** Measurement of 131I activity in the thyroid of nuclear medical staff.

**Subject**	**Sex**		**Type of contact**				**Results**				
			**Manual dispensing and**		**Automatic dispensing and**		**Number of thyroid**			**Check out the range of** ^**131**^**I**	
			**and manual delivery**		**automatic dosing**		**detected** ^**131**^**I**			**thyroid activity [B q]**	
**Subject**	**Male**	**Female**	**Number**	**The number**	**Number**	**The number**	**Physician**	**Nurse**	**Technician**	**Max**	**Min**
				**of detected**		**of detected**					
2019	9	11	6	0	14	8	6	1	1	1,271.68 ± 279.77	30.03 ± 6.61
2020	18	20	5	0	33	11	4	5	2	461.39 ± 101.51	55.70 ± 12.25
2021	15	17	-	-	32	13	6	4	3	6,070.00 ± 1,335.40	70.90 ± 15.60

**Table 4:** Estimate of internal thyroid ^131^I dose in 2019.

**Professional category**	**Activity (Bq)**	***m*** **(t)**	***e***	**Results (Sv)**	**Thyroid organ dose (mSv)**	**Annual effective dose (mSv)**	**Intake (Bq)**
Physicians	1,097.57 ± 241.47	1.20 x 10^−1^	1.10 x 10^−8^	1.01 x 10^−4^	0.1006	1.2241	9,146.42
Nurse	1,271.68 ± 279.77	1.20 x 10^−1^	1.10 x 10^−8^	1.17 x 10^−8^	0.1166	1.4183	10,597.33
Physicians	180.87 ± 39.80	9.00 x 10^−2^	1.10 x 10^−8^	2.21 x 10^−5^	0.0221	0.2690	2,009.67
Physicians	81.25 ± 17.88	1.20 x 10^−1^	1.10 x 10^−8^	7.45 x 10^−6^	0.0074	0.0906	677.08
Physicians	38.05 ± 6.79	9.00 x 10^−2^	1.10 x 10^−8^	4.65 x 10^−6^	0.0047	0.0566	422.78
Physicians	423.13 ± 93.09	1.2 x 10^−1^	1.10 x 10^−8^	3.88 x 10^−5^	0.0388	0.4719	3,526.08
Physicians	30.03 ± 6.61	6.00 x 10^−3^	1.10 x 10^−8^	5.51 x 10^−5^	0.0551	0.6699	5,005.00
Technician	40.53 ± 8.92	1.20 x 10^−1^	1.10 x 10^−8^	3.72 x 10^−6^	0.0037	0.0452	337.75

**Table 5:** Estimate of internal thyroid ^131^I dose in 2020.

**Professional category**	**Activitye (Bq)**	**m (t)**	***e***	**Results (Sv)**	**Thyroid organ dose (mSv)**	**Annual effective dose (mSv)**	**Intake (Bq)**
Nurse	304.43 ± 66.97	1.20 x 10^−1^	1.10 x 10^−8^	2.79 x 10^−5^	0.0279	0.3395	2,536.92
Physicians	378.79 ± 83.33	1.10 x 10^−1^	1.10 x 10^−8^	3.79 x 10^−5^	0.0379	0.4609	3,443.55
Nurse	55.70 ± 12.25	6.00 x 10^−3^	1.10 x 10^−8^	0.0001	0.1021	1.2424	9,283.33
Nurse	461.39 ± 101.51	1.20 x 10^−1^	1.10 x 10^−8^	4.23 x 10^−5^	0.04230	0.5146	3,844.92
Physicians	253.79 ± 55.83	7.40 x 10^−2^	1.10 x 10^−8^	3.77 x 10^−5^	0.0377	0.4590	3,429.60
Physicians	131.59 ± 28.95	1.20 x 10^−1^	1.10 x 10^−8^	1.21 x 10^−5^	0.0121	0.1468	1,096.58
Nurse	107.96 ± 23.75	5.60 x 10^−2^	1.10 x 10^−8^	2.12 x 10^−5^	0.0212	0.2580	1,927.86
Nurse	135.63 ± 29.84	9.00 x 10^−2^	1.10 x 10^−8^	1.66 x 10^−5^	0.0166	0.2017	1,507.00
Technician	235.37 ± 51.78	7.40 x 10^−2^	1.10 x 10^−8^	3.50 x 10^−5^	0.0350	0.4257	3,180.68
Physicians	99.61 ± 21.91	7.40 x 10^−2^	1.10 x 10^−8^	1.48 x 10^−5^	0.0148	0.1802	1,346.08
Technician	294.61 ± 64.81	5.60 x 10^−2^	1.10 x 10^−8^	5.79 x 10^−5^	0.0579	0.7041	5,260.89

**Table 6:** Estimate of internal thyroid ^131^I dose in 2021.

**Professional category**	**Activitye (Bq)**	**m(t)**	**e**	**Results (Sv)**	**Thyroid organ dose (mSv)**	**Annual effective dose (mSv)**	**Intake (Bq)**
Nurse	4,615.00 ± 1015.30	1.20 x 10^−1^	1.10 x 10^−8^	0.0004	0.4230	5.1470	38,458.33
Physicians	967.00 ± 212.74	8.20 x 10^−2^	1.10 x 10^−8^	0.0001	0.1297	1.5783	11,792.68
Physicians	388.00 ± 87.56	1.20 x 10^−1^	1.10 x 10^−8^	3.56 x 10^−5^	0.0356	0.4327	3,233.33
Physicians	869.00 ± 191.18	1.20 x 10^−1^	1.10 x 10^−8^	7.97 x 10^−5^	0.0797	0.9692	7,241.67
Nurse	5,050.00 ± 1,111.00	1.20 x 10^−1^	1.10 x 10^−8^	0.0005	0.4629	5.6322	42,083.33
Physicians	705.40 ± 155.19	7.40 x 10^−2^	1.10 x 10^−8^	0.0001	0.1049	1.2758	9,532.43
Technician	95.00 ± 20.90	1.20 x 10^−1^	1.10 x 10^−8^	8.71 x 10^−6^	0.0088	0.1060	791.67
Nurse	6,070.00 ± 1,335.40	1.20 x 10^−1^	1.10 x 10^−8^	0.0006	0.5564	6.7697	50,583.33
Technician	446.00 ± 98.12	7.40 x 10^−2^	1.10 x 10^−8^	6.63 x 10^−5^	0.0663	0.8066	6,027.03
Physicians	2,630.00 ± 578.60	1.20 x 10^−1^	1.10 x 10^−8^	0.0002	0.2411	2.9332	21,916.67
Physicians	70.90 ± 15.60	6.00 x 10^−3^	1.10 x 10^−8^	0.0001	0.1300	1.5815	11,816.67
Nurse	195.30 ± 42.97	5.60 x 10^−2^	1.10 x 10^−8^	3.84 x 10^−5^	0.0384	0.4667	3,487.50
technician	86.90 ± 19.12	6.00 x 10^−3^	1.10 x 10^−8^	0.0002	0.1593	1.9384	14,483.33

## Discussion

In the diagnosis and treatment of nuclear medicine, ^90m^Tc, ^131^I, and ^18^F are frequently used, of which ^131^I is the most widely used in clinical practice and is highly volatile. Improper operation and protection will easily cause serious pollution in the workplace, which will lead to internal irradiation of the staff. Radiation sources in nuclear medicine mainly come from three aspects: (1) radiopharmaceuticals, including dispensing kits, generators, and therapeutic nuclides; (2) work waste, including needles, cotton balls, and laboratory supplies; (3) the patient and the feces of the patient. In the first two cases, management should be strengthened, and special personnel are responsible for the use of records. The patient is a mobile source of radiation, and the education of the patient is also very important (8–11, 13).

The internal radiation monitoring of nuclear medicine workers, especially the monitoring of ^131^I, requires urgent attention.

The International Atomic Energy Agency (IAEA) proposed in 1999 the principles of determining intra-staff radiation monitoring for nuclear medicine on the basis of studies by relevant countries. Chile, Portugal, and other countries conducted a survey of the nuclear medical staff who directly manipulated nuclides based on the above-mentioned decision-making principles. The results showed that about 70% of the staff who directly manipulated nuclides in the nuclear medical disciplines needed internal radiation monitoring. Internal irradiation monitoring is mainly carried out through air sampling, *in vitro* direct measurement, biological sample analysis, and other methods.

Internal radiation monitoring and dose estimation should be carried out for nuclear medicine workers. Internal radiation monitoring is mainly carried out through methods such as air sampling, *in vitro* direct measurement, biological sample analysis (DNA strand breaks in leukocytes of peripheral blood), and other methods (14–19).

Recommended dose estimation method by ICRP and GBZ 129-2016 and related occupational dose software, with the advent of the use of radioactive sources in various fields such as health physics, industry, energy, and environmental applications, nuclear radiation detectors have become the most fundamental instruments. NaI detectors are one of the traditional methods for measuring iodine in the thyroid. NaI (T1) scintillation survey meters use counter devices, which have disadvantages such as low detection efficiency, poor positioning, large measurement errors, and more radioactive iodine intake. The commonly used traditional gamma spectroscopic analysis tools still use the classical method based on peak search and matching. In the calibration phase of the peaks, there are usually overlaps due to the poor resolution in the NaI detector spectrum, and it is hard to accurately detect the high background and great fluctuation. How to effectively subtract the background is one of the key process technologies of spectroscopic data analysis in gamma-ray energy spectra measurement.

To overcome these issues, we have developed a new method based on the 2-in^*^2-in NaI (Tl) scintillation spectrometer. The 2-in^*^2-in NaI (Tl) scintillation spectrometer has the advantages of higher speed and higher energy resolution.

In this study, in 3 consecutive years, from 2019 to 2021, the thyroid activity of ^131^I was measured in members of nuclear medicine departments. According to the GBZ 128, Specifications for individual monitoring of occupational external radiation in 2021, the annual dose was higher than 5 mSv for three staff members of the studied cohort (9.4%), indicating that all radiation workers engaged in iodine therapy in the nuclear medicine department should conduct internal radiation dose monitoring. According to the monitoring data for 3 consecutive years, 3 cases were detected whose workload was significantly higher than that of other workers because of their high frequency of drug administration to patients and their longer cumulative working hours. The type of contamination depended significantly on the type of occupational exposure. Nursing staff engaged in iodine therapy are the most contaminated population because they are directly involved with the preparation, packaging, and management of radiopharmaceuticals and are needed at every stage of treatment. Nuclear medicine workers who were involved with the manual drug packaging and delivery had the highest ^131^I activity in the thyroid, while staff members involved with the automatic packaging and drug delivery were not found with ^131^I activity. It is suggested that radiation workers engaged in iodine treatment should arrange appropriate workloads to reduce the dose derived from the exposure to ^131^I.

Nevertheless, the estimated dose of ^131^I to the thyroid gland is lower than the average annual effective dose limit for radiation workers, which is 20 mSv per year. Because the thyroid is radiation-sensitive, under the linear no-threshold theory of radiation carcinogenesis, the thyroid dose should be reduced. In addition, as radiation workers are exposed to external radiation, more attention needs to be paid to their occupational health. Nuclear medicine personnel who work with ^131^I have some degree of internal exposure. This distribution pattern can be explained by the types of duties performed with respect to exposure to ^131^I. Nurses, radiology technicians, and radiopharmacists are the most contaminated population because they are directly involved in the preparation, packaging, and management of radiopharmaceuticals, which are needed at every stage of treatment. In contrast, nuclear medicine workers who manually package and deliver ^131^I had the highest ^131^I activity in the thyroid. Staff involved with the automatic packaging and drug delivery showed no ^131^I activity.

At present, the monitoring of domestic nuclear medicine workers is limited to the measurement of the individual dose due to external irradiation and the distribution ratio of the annual per capita dose exceeding 5 mSv, which is higher than that of other radiation workers. Although the estimated ^131^I dose to the thyroid is below the average effective dose limit of 20 mSv per year for radiation workers, the incidence of posterior polar subcapsular opacification and chromosome unstable aberration in peripheral blood due to ionizing radiation in nuclear medicine workers is significantly higher than that of other occupational irradiated groups. In the research of nuclear medicine occupational radiation protection in China, there are few reports on internal radiation monitoring methods and internal radiation dose of workers.

The health of staff and the safety of patients are paramount, and protective equipment and instruments are indispensable. When building or rebuilding a new nuclear medical facility, the installation of protective equipment and the rational and scientific layout of departments should be taken into consideration. The corridors for patients and staff should be separated.

In carrying out daily work and research experiments with large doses, it is very important to design and prepare in advance. Unskilled and unreasonable operation procedures will prolong the time of exposure to radioactive materials and bring unnecessary radiation exposures, leading to higher radiation doses. Radiation exposure in nuclear medicine is mainly due to unavoidable exposure and radiation contamination in the department. However, due to the lack of systematic radiological protection education and the “invisibility” of radiation, personal protection awareness is crucial to avoid radioactive pollution in the department.

## Conclusion

In this article, a simple monitoring and evaluation method for ^131^I in the thyroid glands of nuclear medicine staff was introduced in detail, in 3 consecutive years from 2019 to 2021, the thyroid ^131^I activity was measured, and the dose was estimated for 3 consecutive years in the nuclear medicine department. ^131^I activity was the highest in the thyroid of nuclear medicine workers involved with the manual packaging and manual delivery of the radiopharmaceutical. No activity was detected in staff involved with the automatic packaging and drug delivery; it was found that there are ^131^I internal exposure occupational risks. Automatic packaging and drug delivery are the best solutions.

Therefore, regular and systematic monitoring of internal contamination should be incorporated into the radiation protection standards of teams dealing with high levels of radioactivity from ^131^I. When radiopharmaceuticals are used for medical treatment or diagnostic purposes, appropriate radiological safety rules should be observed, and appropriate safety measures should be taken.

## Data availability statement

The original contributions presented in the study are included in the article/supplementary material, further inquiries can be directed to the corresponding author.

## Ethics statement

The studies involving human participants were reviewed and approved by Gansu Provincial Center for Disease Control and Prevention. The patients/participants provided their written informed consent to participate in this study.

## Author contributions

XZ revised the grammar of the manuscript. GL and YeL were a major contributor in writing the manuscript. All authors were involved in conceptual conception, protocol formulation, data collection and analysis, occupational health monitoring of radiation workers, manuscript writing, read, and approved the final manuscript.

## Funding

This study has received funding from the Lanzhou Science and Technology Bureau (2018-1-127), Gansu Province Natural Science Foundation (21JR7RA653), and Gansu Provincial Health Commission (GSWSKY2018-48).

## Conflict of interest

The authors declare that the research was conducted in the absence of any commercial or financial relationships that could be construed as a potential conflict of interest.

## Publisher's note

All claims expressed in this article are solely those of the authors and do not necessarily represent those of their affiliated organizations, or those of the publisher, the editors and the reviewers. Any product that may be evaluated in this article, or claim that may be made by its manufacturer, is not guaranteed or endorsed by the publisher.
